# The association of minerals intake in three meals with cancer and all-cause mortality: the U.S. National Health and Nutrition Examination Survey, 2003–2014

**DOI:** 10.1186/s12885-021-08643-5

**Published:** 2021-08-11

**Authors:** Xiaoqing Xu, Wei Wei, Jiaxu Xu, Jiaxin Huang, Li Li, Tianshu Han, Jiayue Qi, Changhao Sun, Ying Li, Wenbo Jiang

**Affiliations:** 1 Department of Nutrition and Food Hygiene, the National Key Discipline, School of Public Health, Harbin Medical University, 157 Baojian Road, Harbin, People’s Republic of China 150081; 2 Department of Postgraduate, Harbin Medical University Cancer Hospital, No.150, Haping Road, Nangang District, Harbin, People’s Republic of China; 3 Department of Endocrinology, The Second Affiliated Hospital of Harbin Medical University, Harbin, People’s Republic of China

**Keywords:** Chrono-nutrition, Minerals, Cancer mortality, NHANES

## Abstract

**Background:**

Intake time of diet has recently been demonstrated to be associated with the internal clock and circadian pattern. However, whether and how the intake time of minerals would influence the natural course of cancer was largely unknown.

**Methods:**

This study aimed to assess the association of mineral intake at different periods with cancer and all-cause mortality. A total of 27,455 participants aged 18–85 years old in the National Health and Nutrition Examination Survey were recruited. The main exposures were the mineral intakes in the morning, afternoon and evening, which were categorized into quintiles, respectively. The main outcomes were mortality of cancer and all causes.

**Results:**

During the 178,182 person-years of follow-up, 2680 deaths, including 601 deaths due to cancer, were documented. After adjusting for potential confounders, compared to the participants who were in the lowest quintile(quintile-1) of mineral intakes at dinner, the participants in the highest quintile intake(quintile-5) of dietary potassium, calcium and magnesium had lower mortality risks of cancer (HRpotassium = 0.72, 95% CI:0.55–0.94, *P* for trend = 0.023; HRcalcium = 0.74, 95% CI:0.57–0.98, *P* for trend = 0.05; HRmagnesium = 0.75, 95% CI:0.56–0.99, *P* for trend = 0.037) and all-cause (HRpotassium = 0.83, 95% CI:0.73–0.94, *P* for trend = 0.012; HRcalcium = 0.87, 95% CI:0.76–0.99, *P* for trend = 0.025; HRmagnesium = 0.85, 95% CI:0.74–0.97, *P* for trend = 0.011; HRcopper = 0.80, 95%CI: 0.68–0.94, *P* for trend = 0.012). Further, equivalently replacing 10% of dietary potassium, calcium and magnesium consumed in the morning with those in the evening were associated with lower mortality risk of cancer (HRpotassium = 0.94, 95%CI:0.91–0.97; HRcalcium = 0.95, 95%CI:0.92–0.98; HRmagnesium = 0.95, 95%CI: 0.92–0.98).

**Conclusions:**

This study demonstrated that the optimal intake time of potassium, calcium and magnesium for reducing the risk of cancer and all-cause mortality was in the evening.

**Supplementary Information:**

The online version contains supplementary material available at 10.1186/s12885-021-08643-5.

## Background

Nowadays, people are more and more interested in when to eat, besides what to eat. The emerging nutritional research field of chrono-nutrition also emphasized that the content, amount and the intake time of food are equally critical for maintaining health [1], and it has been demonstrated that eating at the wrong time may lead to circadian desynchronization, which further negatively impacted on their health [2–4]. An increasing amount of rodent and human studies have found that the intake time of energy and macronutrient had an impact on the internal clock and circadian patterns that were related to the development of cancer [5–9]. However, it is largely unknown whether and how the intake time of micronutrients would influence the natural course of cancer.

Minerals are essential components of micronutrients for humans [10], which not only can make up human tissues but also maintain normal physiological functions of the human body [11]. A large number of population studies have shown that dietary intake of different minerals may contribute to the development of various cancer. In the Rotterdam Study, dietary zinc and iron intake are associated with reduced lung cancer risk [12]. A case-control study in Taixing showed that increased dietary intake of selenium and zinc may decrease the risk of squamous cell carcinoma of the esophagus in a low-selenium area of China [13]. Other studies have shown that both selenium and magnesium have a protective effect against cancer, and dietary intake of zinc and copper has been linked to a lower risk of lung cancer [14–17]. A high proportion of dietary potassium and sodium had a statistically significant protective effect on colon and rectal cancer in women [18]. Total calcium intake increases the risk of total prostate cancer [19]. Increased dietary intake of calcium, magnesium, and potassium may help reduce the risk of colorectal cancer, while increased dietary iron intake may increase the risk of colorectal cancer [20]. Total dietary iron intake may be associated with an increased risk of breast cancer, in particular through lipid peroxidation [21]. Besides, numerous animal studies have shown that minerals were identified as involved in many homeostatic mechanisms related to cancer, such as oxidative stress, inflammation response, specific immunity, carcinogenesis and malignant cell proliferation [22–28]. Recently, rodent researchers also found that some minerals were components of the mammalian biological clock and were identified as regulators of circadian rhythm-dependent metabolism [29–33]. Based on these pieces of evidence, we therefore hypothesized that the right and regular mineral intake time is essential for regulating the body’s metabolism and preventing the process of cancer. Accordingly, this study prospectively examined the association between the mineral intake time and cancer and all-cause mortality using the representative sample of the non-institutionalized civilian population in the U.S. from the National Health and Nutrition Examination Survey (NHANES).

## Methods

### Study population

The data reported in the present study come from NHANES collected from 2003 to 2014 (NHANES 2003–2014). The NHANES is a program of studies designed to determine the association of nutritional status with health promotion and disease prevention in the United States, which combines interviews and physical examinations. The interview includes demographic, dietary, socioeconomic, and health-related questions. The examination component consists of medical, dental, and physiological measurements, as well as laboratory tests. The NHANES used a stratified, multistage study (from counties or small groups of contiguous counties to individuals within a household) to recruit participants to identify a nationally representative sample of the non-institutionalized household population of the U.S. The survey examines a nationally representative sample of approximately 5000 people each year, who are distributed in counties across the country. And the data reported in the present study come from NHANES collected from 2003 to 2014 (NHANES 2003–2014). Before the analysis, we excluded participants who did not consume dietary minerals within a day. In our study, we conducted prospective analyses on adult participants (age ≥ 18 years & age ≤ 85 years) in NHANES from 2003 to 2014. A total of 27,455 participants, including 13,359 men and 14,096 women, were included in the present study, after excluding those who had missing information on any dietary intake and mortality; and those whose total energy intake > 5000 kcal or < 500 kcal in on the day.

### Dietary assessment

The dietary intake of essential micronutrients among the subjects was assessed using 24-h dietary recalls during two typical, non-consecutive days for each participant. The first data was completed in person while the second by telephone. The dietary intakes of foods were verified with the data collected by a validated semi-quantitative food frequency questionnaire (FFQ). The dietary intake components are integrated into the 36 main food groups of MyPyramid based on the MyPyramid equivalent database 2.0 (MPED 2.0) of the U.S. Department of Agriculture Survey Food. All dietary estimates were assigned by using the guidelines of the U.S. Department of Agriculture’s Food and Nutrient Database for Dietary Studies. In brief, the daily dietary intakes of minerals were calculated by combining serving frequency per day, the average amount of serving, and portion per unit. And according to intake time, the intakes of nutrients are divided into breakfast, lunch, dinner and snacks (between breakfast and lunch, between lunch and dinner and after dinner).

### Main exposure

The exposure variable that defines the main exposure could be set as the intake of these minerals at breakfast, lunch and dinner, respectively. The following minerals which have been proven to be associated with cancer [12–21] were studied in the present study: potassium, calcium, sodium, magnesium, selenium, zinc, iron, copper and phosphorus. The sum of breakfast and snack before lunch (< 12:00 p.m.), lunch and snack after lunch (12:00 p.m.-18:00 p.m.), dinner and snack after dinner (> 18:00 p.m.) were identified as the intake of minerals in the morning, afternoon, and evening, respectively.

### Main outcome

This study that analyzed mortality outcomes depend on the US National Death Index (NDI) to ascertain vital status. NDI is a highly reliable database containing information from death certificates that has achieved widespread use among health and medical investigators (https://www.cdc.gov/nchs/data-linkage/mortality.htm). All-cause mortality refers to the total number of deaths caused by various causes in a certain period, including the diseases of heart, malignant neoplasms, chronic lower respiratory diseases, accidents (unintentional injuries), cerebrovascular diseases, Alzheimer’s disease, diabetes mellitus, influenza and pneumonia, nephritis, nephrotic syndrome and nephrosis, intentional self-harm (suicide), chronic liver disease and cirrhosis, septicemia and assault (homicide). The documents related to the NDI mortality could be accessed at https://www.cdc.gov/nchs/data-linkage/mortality.htm, https://www.cdc.gov/nchs/nvss/mortality/lcwk9.htm. And the death from cancer was defined as ICD-10 codes I19-I43. A total of 2680 deaths were available for further analysis, and of these, 601 deaths were due to cancer.

### Confounding and effect modification measurements

The covariates were set as follows: (1)socio-demographic: age (continuous), sex (male or female), race/ethnicity (non-Hispanic white/non-Hispanic black/Mexican American/other), education level (< 9th grade, 9th–11th grade, high school graduate, general educational development (GED) or equivalent, some college or Associate in Arts degree, or college graduate or above), annual household income (< $20,000, $20,000 - $45,000, $45,000 - $75,000, $75,000 - $100,000, or > $100,000); (2) health behaviors: regular exercise (yes/no), current smoker (yes/no), current drinker (yes/no); (3) physical examination: body weight and height were measured with a standard medical balance and used to calculate the BMI (kg/m^2^); (4) dietary habits factors: total energy (kcal/day), total dietary consumption corresponding to specific dietary mineral intake (g/day); breakfast skipping (yes/no); dietary supplement use (yes/no); and diet quality. Diet quality was calculated by the Alternative Healthy Eating Index (AHEI), which is based on foods and nutrients predictive of chronic disease risk and was associated inversely with chronic disease risk [34, 35].

### Statistical analysis

The data were checked for completeness, cleaned and analyzed using R 4.0.0 software according to the survey data commands, and then a 5% significance level was considered. Socio-demographic, dietary nutrient intake and health-risk lifestyle factors were expressed as relative frequencies. For categorical variables, frequencies and percentages would be presented. For continuous variables, mean and standard deviation were reported. The total minerals in breakfast, lunch and dinner were divided into quintiles based on distribution, respectively. General linear models after adjusting for age and the Chi-square test were used to compare baseline characteristics by mortality status. All statistical analyses were performed by R 4.0.0, and two-sided *P* < 0.05 was considered to be statistically significant.

### Cox proportional hazards models

The Cox proportional-hazards (CPH) regression model has been widely used to analyze time-to-event data with censoring and covariates. In this study, it was used to determine cancer and all-cause mortality, and the results were presented as hazard ratios (HRs) with 95% confidence intervals (CIs). Follow-up survival time was calculated by person-months from the date of the NHANES interview date until the date of death or censoring on December 31, 2015, whichever came first. Important potential confounding variables were often included and controlled in all CPH models, such as age, sex, ethnicity, income, education level, regular exercise, smoking and drinking status, BMI, the prevalence of diabetes, hypertension, hyperlipidemia, nutrient supplement use, AHEI, total intake of energy, specific dietary minerals intake. In this study, the specific mineral intakes across a day were estimated in the same model.

### Predicted equivalent minerals substitution models

Food substitutions model is used to study the relationship between food or nutrient substitution and related diseases or health outcomes, and to provide dietary recommendations for disease prevention and treatment [36]. At present, many studies have adopted substitution analysis to replace one food or nutrient with another food or nutrient under the condition of equal energy or equal intake, and then observe the changes of epidemiological indicators [37, 38]. Based on the CPH model above, we established an equivalent mineral replacement model to evaluate the changes in mortality risk of cancer by switching the intake of one mineral at a one-time period to another time period. In the present study, we equivalently converted 10% of the intake of potassium, calcium and magnesium from morning or afternoon to evening to observe whether and how the risk of death from cancer changes.

### Sensitivity analysis

Five sets of sensitivity analyses were additionally performed to assess the robustness of the results. In set 1, the association of total dietary minerals intake with cancer and all-cause mortalities was performed to examine whether the intake time could provide more information than the total amount of mineral intake. In set 2, we excluded the participants without breakfast consumption to eliminate the effect of breakfast skipping. In set 3, we removed participants with appropriate mineral supplements to minimize the impact of mineral supplements on results. In set 4, we excluded the participants who had a follow-up time of fewer than two years (including those who died within two years of follow-up) to minimize potential reverse causation caused by severe illness, such as cardiovascular disease or cancer. In set 5, we corrected the total dietary mineral intake in one day. Besides, to exclude the effect of other minerals, we established a score of mineral intakes and then re-analyzed the association of time-period intakes of a particular mineral on mortalities.

## Results

### Baseline characteristics

The demographic and nutrition characteristics in terms of the survival status of cancer were presented in Table 1. Compared with other participants, the participants who died due to cancer were more likely to be older, men, and non-Hispanic white and had higher education, income, dietary supplements taken, the prevalence of hypertension, diabetes, and dyslipidemia, and lower BMI, exercise, total energy intake, total calcium intake, and total magnesium intake (*P* < 0.05). Other variables, including smoking, breakfast skipping, AHEI, total potassium intake, were not significantly different.

**Table 1:** The baseline characteristics of studying variables by mortality status

Variables	Other participants (***N*** = 26,854)	Death due to cancer (***N*** = 601)	***P***-value
Age (years)	48.8 (18.8)	67.9 (13.1)	< 0.001
Men (%)	12,997 (48.4)	358 (59.6)	< 0.001
Non-Hispanic white (%)	12,568 (46.8)	330 (54.9)	< 0.001
Current smoking (%)	6714 (25.0)	136 (22.6)	0.660
Current drinking (%)	17,160 (63.9)	392 (65.2)	0.004
College graduate or above (%)	13,508 (50.3)	362 (60.3)	< 0.001
>$ 100,000 annual household income (%)	5639 (21.0)	185 (30.8)	< 0.001
BMI (kg/m^2^)	28.7 (6.7)	28.4 (6.1)	0.011
Regular exercise (%)	6311 (23.5)	110 (18.3)	0.03
Dietary supplement use (%)	13,185 (49.1)	346 (57.6)	< 0.001
Hypertension (%)	11,010 (41.0)	385 (64.1)	< 0.001
Diabetes (%)	4109 (15.3)	160 (26.6)	< 0.001
Dyslipidemia (%)	8566 (31.9)	254 (42.3)	< 0.001
Total energy intake (kcal/d)	2048.5 (793.6)	1825.6 (712.9)	< 0.001
Total calcium intake (g/d)	913.4 (485.7)	804.2 (450.5)	0.036
Total potassium intake (g/d)	2630.5 (1052.9)	2527.4 (1061.3)	0.864
Total magnesium intake (g/d)	288.8 (123.6)	262.3 (112.3)	0.041
AHEI	52.7 (13.5)	50.3 (13.3)	0.549
Breakfast skipping (%)	1611 (6.0)	30 (5.0)	0.382
Dietary Supplements Taken (%)	13,185 (49.1)	346 (57.6)	< 0.001

Continuous variables are presented as mean (standard deviation). Categorical variables are presented as frequencies (percentages)

Hypertension was defined by a self-reported diagnosis, the systolic blood pressure ≥ 140 mmHg, or the diastolic blood pressure ≥ 90 mmHg

Hyperlipidemia was defined as serum triglyceride≥2.26 mmol/L, or serum cholesterol≥6.22 mmol/L, or low-density lipoprotein≥4.14 mmol/L.

Diabetes was defined by a self-reported diagnosis, anHbA1c level ≥ 6.5%, or a fasting plasma glucose level ≥ 7.0 mmol/L.

AHEI, Alternative Healthy Eating Index

### Cox proportional hazards models

**Fig. 1:**
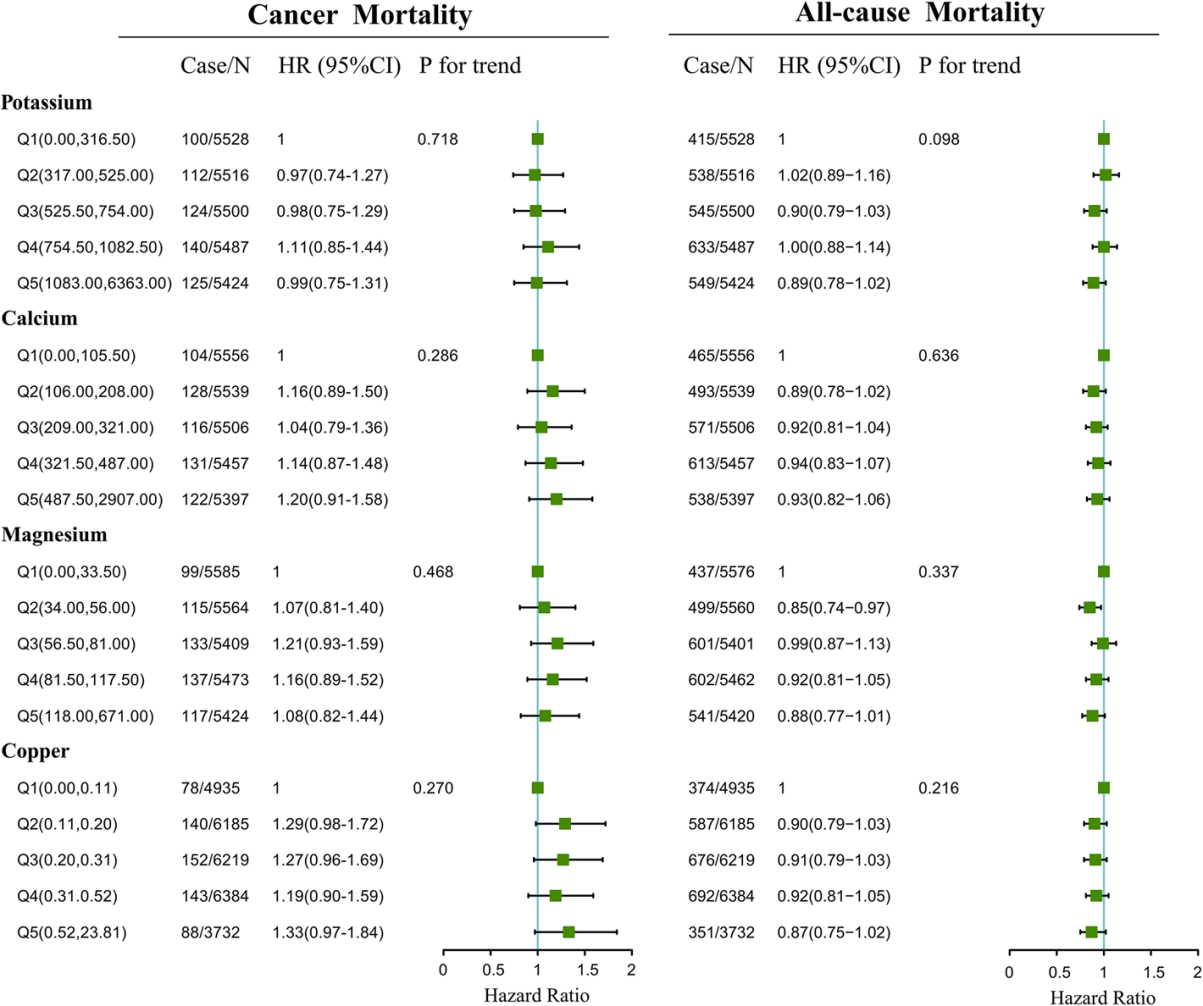
Multivariate adjusted HRs of the dietary potassium, calcium and magnesium intake at breakfast with cancer and all-cause mortality. Adjustments included age, sex, ethnicity, income, education level, regular exercise, smoking and drinking status, BMI, the prevalence of diabetes, hypertension, hyperlipidemia, nutrient supplement use, AHEI, total intake of energy, specific dietary minerals intake. Case/N, number of case participants/total; Q, Quintile; AHEI, Alternative Healthy Eating Index

**Fig. 2:**
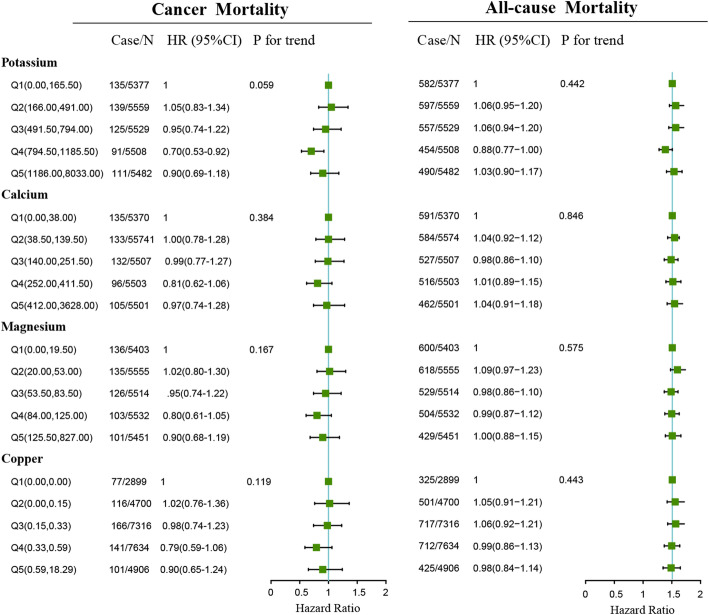
Multivariate adjusted HRs of the dietary potassium, calcium and magnesium intake at lunch with cancer and all-cause mortality. Adjustments included age, sex, ethnicity, income, education level, regular exercise, smoking and drinking status, BMI, the prevalence of diabetes, hypertension, hyperlipidemia, nutrient supplement use, AHEI, total intake of energy, specific dietary minerals intake. Case/N, number of case participants/total; Q, Quintile; AHEI, Alternative Healthy Eating Index

**Fig. 3:**
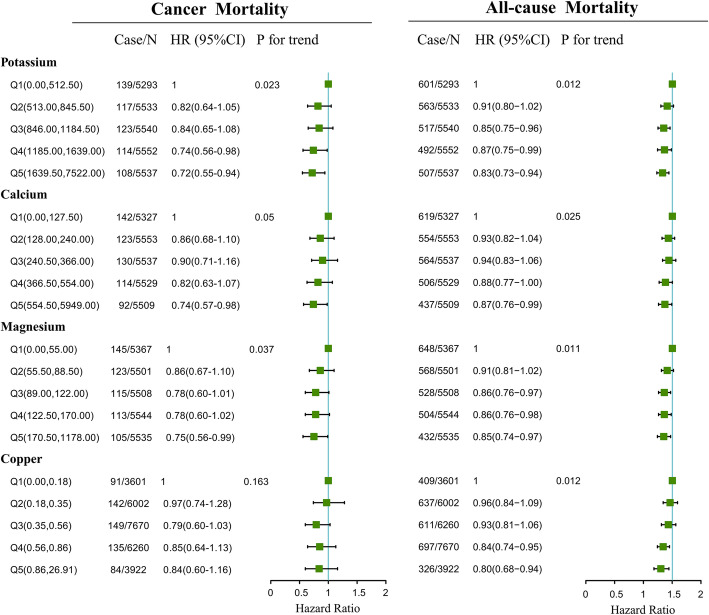
Multivariate adjusted HRs of the dietary potassium, calcium and magnesium intake in the evening with cancer and all-cause mortality. Adjustments included age, sex, ethnicity, income, education level, regular exercise, smoking and drinking status, BMI, the prevalence of diabetes, hypertension, hyperlipidemia, nutrient supplement use, AHEI, total intake of energy, specific dietary minerals intake. Case/N, number of case participants/total; Q, Quintile; AHEI, Alternative Healthy Eating Index

Association of dietary potassium, calcium, magnesium and copper consumed in the morning, afternoon and evening with mortality of cancer and all-cause is presented in Fig. 1 to Fig. 3. Association of other kinds of mineral intakes in the morning, afternoon, and evening with mortality of cancer and all-cause was presented in Table 2. Besides, we found the relative intake of minerals in different periods across a day was different. The distributions of mineral intake were presented in Supplementary Table 1. In the morning and afternoon (Fig. 1 and Fig. 2), no significant association of 9 mineral intakes with mortality of cancer and all-cause was observed. In the evening (Fig. 3), as indicated by HR and 95% CI, compared to the participants in the lowest quintile of mineral intakes, participants in the highest quintile intakes of potassium, calcium and magnesium at dinner had lower mortality risks of cancer (HR = 0.72, 95% CI:0.55–0.94, *P* for trend = 0.023; HR = 0.74, 95% CI:0.57–0.98, *P* for trend = 0.05; HR = 0.75, 95% CI:0.56–0.99, *P* for trend = 0.037) and potassium, calcium, magnesium and copper at dinner had lower all-cause mortality (HR = 0.83, 95%CI: 0.73–0.94, *P* for trend = 0.012; HR = 0.87, 95%CI: 0.76–0.99, *P* for trend = 0.025; HR = 0.85, 95%CI: 0.74–0.97, *P* for trend = 0.011;HR = 0.80, 95%CI: 0.68–0.94, *P* for trend = 0.012).

**Table 2:** Adjusted HRs for dietary sodium, selenium, zinc, phosphorus and iron at breakfast, lunch and dinner and cancer, and all-cause mortality

	Cancer mortality		All-cause mortality	
	Case/N	HR (95%CI)	Case/N	HR (95%CI)
**Sodium (breakfast)**				
Q1(0.00,241.00)	96/5554	1	415/5554	1
Q2(241.50,451.00)	127/5515	1.26 (0.96–1.66)	573/5515	1.04 (0.92–1.19)
Q3(451.50,706.00)	118/5477	1.07 (0.81–1.42)	581/5477	0.97 (0.85–1.10)
Q4(706.50,1104.00)	130/5480	1.15 (0.88–1.52)	610/5480	1.03 (0.90–1.17)
Q5(1104.50,7315.50)	130/5429	1.31 (0.99–1.73)	501/5429	1.02 (0.89–1.18)
*P* for trend		0.194		0.843
**Sodium (lunch)**				
Q1(0.00,133.50)	133/5372	1	573/5372	1
Q2(134.00,675.00)	142/5556	1.10 (0.86–1.40)	614/5556	1.07 (0.95–1.21)
Q3(676.00,1136.50)	125/5500	1.04 (0.81–1.34)	557/5500	1.07 (0.95–1.22)
Q4(1137.00,1732.00)	104/5505	0.84 (0.64–1.10)	496/5505	0.98 (0.86–1.12)
Q5(1732.50,7313.50)	97/5522	0.85 (0.64–1.13)	440/5522	1.01 (0.88–1.15)
*P* for trend		0.074		0.656
**Sodium (dinner)**				
Q1(0.00,661.00)	148/5305	1	645/5305	1
Q2(661.50,1140)	135/5524	0.93 (0.73–1.19)	599/5524	0.96 (0.85–1.08)
Q3(1140.50,1617.50)	113/5520	0.76 (0.60–1.00)	514/5520	0.88 (0.78–1.00)
Q4(1618.00,2304.50)	100/5541	0.72 (0.55–0.96)	504/5541	0.92 (0.81–1.05)
Q5(2305.00,12,776.00)	105/5565	0.76 (0.57–1.01)	418/5565	0.87 (0.75–1.01)
*P* for trend		0.010		0.040
**Selenium (breakfast)**				
Q1(0.00,11.40)	130/7640	1	603/7640	1
Q2(11.40,19.20)	111/4962	1.16 (0.90–1.50)	500/4962	0.95 (0.84–1.07)
Q3(19.20,28.60)	139/4999	1.44 (1.13–1.83)	561/4999	1.07 (0.95–1.21)
Q4(28.60,42.50)	114/4941	1.08 (0.84–1.41)	585/4941	1.01 (0.90–1.14)
Q5(42.50,509.25)	107/4913	1.24 (0.95–1.62)	431/4913	0.99 (0.87–1.13)
*P* for trend		0.182		0.725
**Selenium (lunch)**				
Q1(0.00,5.55)	163/6509	1	693/6509	1
Q2(5.55,22.10)	135/5536	1.04 (0.82–1.31)	623/5536	1.10 (0.98–1.23)
Q3(22.15,35.65)	112/5126	0.95 (0.74–1.22)	504/5126	1.01 (0.90–1.14)
Q4(35.65,54.70)	101/5144	0.92 (0.71–1.19)	440/5144	0.97 (0.85–1.10)
Q5(54.75,888.95)	90/5140	0.88 (0.67–1.16)	420/5140	1.03 (0.90–1.17)
*P* for trend		0.294		0.728
**Selenium (dinner)**				
Q1(0.00,21.90)	152/5938	1	713/5938	1
Q2(21.90,37.30)	115/5339	0.88 (0.68–1.13)	552/5339	0.94 (0.84–1.06)
Q3(37.30,53.65)	106/5385	0.85 (0.66–1.10)	493/5385	0.89 (0.79–1.01)
Q4(53.65,78.00)	123/5388	1.04 (0.80–1.34)	506/5388	1.04 (0.92–1.17)
Q5(78.05,615.20)	105/5405	0.94 (0.71–1.24)	416/5405	0.94 (0.82–1.08)
*P* for trend		0.973		0.773
**Zinc (breakfast)**				
Q1(0.00,1.04)	100/5994	1	473/5994	1
Q2(1.05,2.34)	175/7654	1.27 (0.99–1.62)	733/7654	0.95 (0.84–1.07)
Q3(2.34,4.25)	157/7219	1.21 (0.85–1.42)	749/7219	0.95 (0.85–1.08)
Q4(4.25,6.54)	98/3814	1.43 (1.07–1.90)	403/3814	1.01 (0.87–1.16)
Q5(6.54,67.97)	71/2774	1.29 (0.94–1.77)	322/2774	0.97 (0.84–1.13)
*P* for trend		0.084		0.909
**Zinc (lunch)**				
Q1(0.00,0.00)	77/2939	1	325/2939	1
Q2(0.01,1.45)	155/6429	0.98 (0.76–1.32)	686/6429	1.07 (0.94–1.23)
Q3(1.45,3.70)	201/8916	0.96 (0.74–1.26)	862/8916	1.02 (0.90–1.17)
Q4(3.70,6.10)	89/5422	0.72 (0.53–1.00)	469/5422	1.01 (0.87–1.17)
Q5(6.10,151.99)	79/3749	0.97 (0.69–1.35)	338/3749	1.06 (0.90–1.25)
*P* for trend		0.234		0.973
**Zinc (dinner)**				
Q1(0.00,2.21)	149/6095	1	678/6095	1
Q2(2.22,4.47)	178/8256	0.91 (0.73–1.14)	793/8256	0.95 (0.85–1.06)
Q3(4.48,6.40)	106/5256	0.85 (0.65–1.11)	495/5256	0.97 (0.85–1.09)
Q4(6.40,9.35)	98/4456	0.94 (0.71–1.23)	428/4456	0.99 (0.87–1.13)
Q5(9.35,166.43)	70/3392	0.86 (0.63–1.19)	286/3392	0.92 (0.78–1.07)
*P* for trend		0.449		0.524
**Phosphorus (breakfast)**				
Q1(0.00,150.00)	88/5548	1	433/5548	1
Q2(150.50,266.00)	130/5525	1.34 (1.02–1.76)	535/5525	0.94 (0.82–1.07)
Q3(266.50,386.50)	140/5503	1.35 (1.02–1.77)	609/5503	0.98 (0.86–1.11)
Q4(387.00,564.00)	124/5476	1.19 (0.90–1.58)	580/5476	0.93 (0.81–1.05)
Q5(564.50,3297.00)	119/5403	1.22 (0.92–1.63)	523/5403	0.94 (0.82–1.07)
*P* for trend		0.484		0.350
**Phosphorus (lunch)**				
Q1(0.00,70.00)	143/5371	1	594/5371	1
Q2(70.50,251.50)	133/5562	0.91 (0.71–1.15)	600/5562	1.04 (0.92–1.17)
Q3(252.00,407.00)	118/5507	0.84 (0.65–1.08)	555/5507	0.99 (0.88–1.12)
Q4(407.50,610.50)	108/5504	0.79 (0.61–1.03)	486/5504	0.96 (0.84–1.09)
Q5(611.00,3280.50)	99/5511	0.80 (0.61–1.06)	445/5511	1.01 (0.89–1.16)
*P* for trend		0.066		0.665
**Phosphorus (dinner)**				
Q1(0.00,265.50)	139/5299	1	642/5299	1
Q2(266.00,432.00)	131/5538	0.91 (0.72–1.17)	564/5538	0.88 (0.78–0.99)
Q3(432.50,606.50)	119/5529	0.86 (0.66–1.11)	562/5529	0.98 (0.87–1.10)
Q4(607.00,844.00)	113/5543	0.78 (0.60–1.02)	483/5543	0.85 (0.75–0.97)
Q5(844.50,4555.00)	99/5546	0.76 (0.56–1.01)	429/5546	0.88 (0.77–1.01)
*P* for trend		0.034		0.074
**Iron (breakfast)**				
Q1(0.00,2.19)	134/7613	1	552/7613	1
Q2(2.19,4.76)	151/7660	1.02 (0.81–1.29)	735/7660	1.07 (0.95–1.19)
Q3(4.76,7.60)	142/5288	1.28 (1.00–1.64)	613/5288	1.14 (1.01–1.29)
Q4(7.60,11.28)	103/3572	1.34 (1.02–1.76)	402/3572	1.06 (0.92–1.21)
Q5(11.28,85.00)	71/3322	0.97 (0.72–1.32)	378/3322	0.97 (0.84–1.11)
*P* for trend		0.276		0.798
**Iron (lunch)**				
Q1(0.00,0.13)	94/3411	1	377/3411	1
Q2(0.13,2.53)	174/7429	0.94 (0.73–1.22)	787/7429	1.06 (0.94–1.21)
Q3(2.53,4.81)	163/7628	0.89 (0.69–1.16)	747/7628	1.07 (0.94–1.22)
Q4(4.81,7.47)	103/5357	0.81 (0.60–1.08)	457/5357	0.96 (0.83–1.11)
Q5(7.47,61.02)	67/3630	0.81 (0.58–1.13)	312/3630	1.05 (0.89–1.23)
*P* for trend		0.109		0.760
**Iron (dinner)**				
Q1(0.00,2.98)	168/6440	1	708/6440	1
Q2(2.98,5.24)	153/7323	0.80 (0.64–1.01)	733/7323	0.94 (0.84–1.04)
Q3(5.24,7.48)	117/5784	0.82 (0.64–1.05)	5,745,784	1.02 (0.91–1.15)
Q4(7.48,10.24)	90/4140	0.79 (0.60–1.04)	358/4140	0.88 (0.76–1.00)
Q5(10.24,97.27)	73/3768	0.79 (0.59–1.07)	307/3768	0.91 (0.78–1.05)
*P* for trend		0.106		0.124

Adjustments included age, sex, ethnicity, income, education level, regular exercise, smoking and drinking status, BMI, prevalence of diabetes, hypertension, hyperlipidemia, nutrient supplement use, AHEI, total daily energy intake and total dietary minerals intake. Q, Quintile. HR, hazard ratio

### Equivalent substitution analysis

**Fig. 4:**
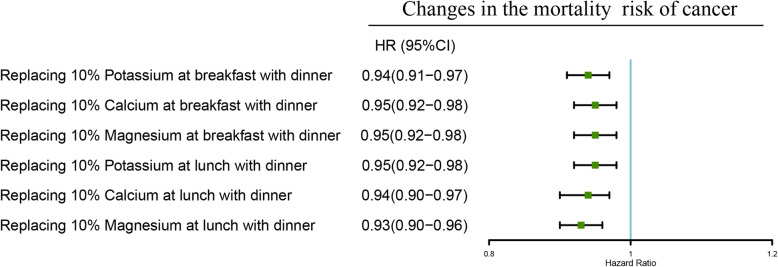
Multivariate adjusted HRs of the intake of the dietary mineral with cancer and all-cause mortality: equivalent potassium, calcium and magnesium substitution from afternoon and evening to morning. Adjustments included age, sex, ethnicity, income, education level, regular exercise, smoking and drinking status, BMI, prevalence of diabetes, hypertension, dyslipidemia, nutrient supplement use, AHEI, total intake of energy, specific dietary minerals intake. AHEI, Alternative Healthy Eating Index

Figure 4 shows the risk of cancer mortality in predicted equivalent substitution models through switching minerals intake in the morning or afternoon to evening. The results showed that HRs for cancer decreased by 6% (HR = 0.94, 95% CI:0.91–0.97), 5% (HR = 0.95, 95% CI: 0.92–0.98), 5% (HR = 0.95, 95% CI: 0.92–0.98) in models with 10% of potassium, calcium and magnesium intake in the morning being equivalently switched to evening. Similarly, the results showed that HRs for cancer reduced by 5% (HR = 0.95, 95% CI: 0.92–0.98), 6% (HR = 0.94, 95% CI: 0.90–0.97), 7% (HR = 0.93, 95% CI: 0.90–0.96) in models with 10% of potassium, calcium and magnesium intake in the afternoon being equivalently switched to evening.

### Sensitivity analysis

In the first set of sensitivity analyze, the total intake of each dietary potassium and calcium was not associated with cancer and all-cause mortality, suggesting that analysis of the intake time could provide more information than amounts of total daily intake only. Besides, total dietary magnesium was associated with reduced mortality risk of cancer, but it became non-significant after correcting the intake time of magnesium, which implied intake-time of magnesium might have a more powerful impact (Supplementary Table 2). After adjustment for breakfast skipping and snack consumption in the morning, the second sensitivity analysis showed that the association between potassium, calcium and cancer mortality outcomes was more significant (Supplementary Table 3). And the third set showed that mineral supplements did not modify the association of mineral intake-time with cancer and all-cause mortality (Supplementary Table 4). In the fourth set of sensitivity analysis, after excluding the participants who died within two years, the association between potassium, calcium and cancer mortality, calcium, magnesium, copper and all-cause mortality outcomes was still significant. In contrast, the association between potassium and all-cause mortality, magnesium and cancer mortality became marginally significant (Supplementary Table 5). In the fifth set of sensitivity analysis, after correcting the total dietary mineral intake in one day, the association between potassium, calcium, magnesium and cancer mortality, magnesium and all-cause mortality outcomes was still significant. However, the association between potassium, calcium, copper and all-cause mortality were non-significant (Supplementary Table 6).

### The food sources of the minerals

Supplementary Table 7 shows the food sources of the minerals analyzed in this study. Fluid milk and calcium-fortified soy milk, intact fruits (whole or cut) excluding citrus, melons, and berries, refined or non-whole grains, other vegetables besides dark green, legumes, total red and orange vegetables, starchy vegetables were the main source of potassium and magnesium in the diet of the study population. The majority of dietary calcium came from fluid milk and calcium-fortified soy milk, refined or non-whole grains, eggs.

## Discussion

This study demonstrated that higher intakes of dietary potassium, calcium and magnesium in the evening were related to lower mortality risks of cancer and all-cause, independent of overall diet quality, total dietary mineral intakes and other traditional risk factors, whereas intakes of them in the morning or afternoon were not associated with mortality outcomes. Further, replacing 10% mineral intake in the morning or lunch with dinner significantly reduced mortality risks of cancer and all-cause.

To the best of our knowledge, this study was the first to examine the association of the intake time of dietary minerals with cancer and all-cause mortality. Our study provided evidence of the potential beneficial effect of high dietary potassium, calcium and magnesium intakes in the evening. It emphasized the importance of the intake time of dietary minerals in a whole day for reducing the mortality risk of cancer. As essential components in foods, minerals may play significant roles in developing many chronic diseases [39, 40]. And numerous epidemiological studies have been performed to evaluate the association between mineral intake and the risk of cancer [41, 42]. However, the results are inconsistent, presumably because the intake time of minerals is not taken into account.

One of the most critical findings in this study is that higher consumption of potassium in the evening was also significantly correlated with lower mortality risks of cancer and all-cause. It is documented that the potassium secretion peaks at night and dietary potassium intake can increase the systemic plasma potassium concentration, which may directly stimulate constant urinary potassium secretion [43]. Therefore, the high intake of potassium in the evening conforms to the circadian rhythm of potassium homeostasis. The resulting coincidence may subsequently promote the natural oscillations of physiologic processes such as glucose, lipid metabolism, and blood pressure. Besides, the circadian rhythm of the clock gene Period 1 (Per1) within the suprachiasmatic nucleus (SCN) is the master pacemaker controlling circadian rhythmicity [44]. And the reduced extracellular concentration of potassium may cause membrane hyperpolarization, thus reversibly abolished the rhythmic expression of Per1 [45, 46]. Hence, it suggests a high intake of potassium in the evening may be helpful for the rhythm expression of circadian clock gene Per1 and sufficient high amplitude.

This study also found that a high intake of calcium in the evening was significantly correlated with reduced mortality risks of cancer and all-cause. The exact biological mechanisms underlying calcium consumption and cancer risk are still not ultimately confirmed. One underlying possible explanation response for the results in this study is that higher calcium intake in the evening might down-regulated the circulating of parathyroid hormone (PTH) [47]. It has been documented that PTH is a tumor promoter acting as a co-mitogen and anti-apoptotic factor, which may further stimulate cell proliferation and intensively suppress apoptosis of cells [48, 49]. The circadian rhythm of PTH showed a biphasic rhythm with a small but significant increase in the late afternoon and a larger, broader increase in the late evening into the early morning with a peak [50]. Thus, we speculated that the higher consumption of calcium in the evening might moderately decrease the secretion of PTH and reduce the chance of malignant proliferation of tumor cells. Another cause suggested that excessive amounts of bile acids in the colonic lumen could be irritating and toxic to the colonic epithelium [51]. Bile acid synthesis has a sharp rise after meals, and the peak occurs from 09:00 to 10:00 p.m. [52]. Hence, a higher calcium intake at dinner may be more likely to induce the conversion of bile acids to insoluble calcium compounds, thereby reducing the adverse effect of bile acids on the colonic epithelium and decreasing the incidence of gastrointestinal tumors.

Furthermore, we found that in addition to calcium and potassium, a high intake of magnesium in the evening was also negatively associated with cancer and all-cause mortality. Previous studies have demonstrated that magnesium is an essential cofactor for some enzyme systems involving DNA repairs such as the repair of nucleotide excision, base excision and mismatch, thereby playing a vital role for DNA synthesis and repair as well as maintain the integrity of DNA [53–55]. Animal studies have shown that the repair capacity of DNA excision in the mouse brain is regulated by circadian rhythm, displaying maximum activity in the afternoon and minimum activity at the midnight [56, 57]. And it is well known that rodent animals and humans have opposite circadian patterns, which means DNA excision repair capacity in humans has an ascending pattern during the night [58]. Therefore, a higher intake of magnesium in the evening is in accord with the rhythm and oscillation of the DNA repair system. Moreover, numerous previous rodent models have shown that magnesium deficiency could induce elevated serum concentrations of inflammatory cytokines and excessive production of free radicals or oxidative stress [59–64]. And several epidemiologic studies have provided some evidence linking magnesium consumption to systemic inflammation and oxidative stress damage, which were explicitly manifested by increased C-reactive protein (CRP) concentration and plasma levels of thiobarbituric acid reactive substances [65, 66]. It has been reported that pro-inflammation cytokines and oxidative stress components had an internal circadian pattern as well, showing the lowest in the afternoon and gradually increasing during the nighttime [67]. Therefore, a higher intake of magnesium at dinner could more likely decrease the amplitude of inflammation and oxidation pattern at night, probably reduced the mortality risk of cancer. In the evening, the cancer mortality of participants in the highest quintile of copper was not significantly different from that of participants in the lowest quintile, but they had lower mortality risks of all-cause mortality. Since all-cause mortality covers deaths caused by various causes within a certain period, it includes deaths caused by not only diseases but also accidents and so on. Therefore, we believe that the significant results of all-cause mortality cannot be used to infer that the high intake of copper at night is better.

According to our results, the optimal intake time of potassium, calcium and magnesium for reducing the risk of cancer and all-cause mortality was in the evening. It is generally believed that the best way to increase your potassium intake is to eat more fruits and vegetables, such as beans, apricots, potatoes, bananas, green leafy vegetables, etc. (www.nal.usda.gov/sites/www.nal.usda.gov/files/potassium.pdf) [68]. The main natural sources of calcium are cheese, tofu, milk (www.nal.usda.gov/sites/www.nal.usda.gov/files/calcium.pdf) [69]; Good sources of magnesium include pumpkin and squash seed kernels, almonds, peanuts, butternuts, unrefined grains (whole), dairy foods, spinach, etc. (www.nal.usda.gov/sites/www.nal.usda.gov/files/magnesium.pdf) [70]. Participants could choose favorite foods for dietary adjustments.

Our study has several important strengths. First, this study was the first to explore the association of mineral intake time with cancer and all-cause mortality. Our findings emphasized that the core of chrono-nutrition, in which the consumption time of minerals should be accorded to the body’s metabolic rhythm for reducing the mortality risks of cancer. Second, NHANES is a nationally representative database based on a probability sample survey design [71]. It provides the most authoritative and comprehensive dietary intake studies in the U.S. as well as the detailed evaluation of lifestyle factors. However, several certain limitations also should be considered. First, since the extent of dietary consumption was derived from 24-h dietary recall methods, we cannot preclude the possibility that dietary measurement error may have obscured meaningful results. Second, we utilized only two dietary measurements over 2 weeks to predict long-term survival in the existing population who were likely to change their eating habits over time. Thus, further studies were necessary to conduct the long-term effect of mineral intake across meals on cancer and all-cause mortality outcomes. Finally, we cannot be sure whether dietary changes including minerals could be made to accomplish the substitution, therefore, we could only urge people to eat the foods rich in potassium, calcium and magnesium at dinner.

## Conclusion

Chrono-nutrition combined elements from nutritional research with chrono-biology, which emphasized the impact of the timing of eating on health outcomes, including mineral intake. This study could provide a theoretical basis for guiding and improving dietary guidelines and individual precision nutrition. In conclusion, the optimal intake time of dietary potassium, calcium and magnesium for reducing the mortality risk of cancer was in the evening.

## Supplementary Information

**Additional file 1 Supplementary Table 1:** The distribution of minerals in each meal. The data in the table is expressed as the mean× 100% (the standard deviation × 100%).

**Additional file 2 Supplementary Table 2:** Multivariate adjusted HRs of the total dietary minerals intake with cancer and all-cause mortality

**Additional file 3 Supplementary Table 3:** Adjusted HRs for the differences in minerals intake between dinner and breakfast and cancer and all-cause mortality with additionally adjusted for breakfast skipping.

**Additional file 4 Supplementary Table 4:** Adjusted HRs for the differences in minerals intake between dinner and breakfast and cancer and all-cause mortality with additionally excluding those having mineral supplements.

**Additional file 5 Supplementary Table 5:** Adjusted HRs for the differences in minerals intake between dinner and breakfast and cancer and all-cause mortality with additionally excluding those who died within two years of follow-up.

**Additional file 6 Supplementary Table 6:** Adjusted HRs for the differences in minerals intake between dinner and breakfast and cancer and all-cause mortality by adjusting the minerals intake score instead of individual mineral intake.

**Additional file 7 Supplementary Table 7:** The food sources of the minerals. The data in the table are expressed as median (minimum, maximum).

## Data Availability

The datasets generated and/or analyzed during the current study are available download from the following website: https://www.cdc.gov/nchs/nhanes/index.htm?CDC_AA_refVal=https%3A%2F%2Fwww.cdc.gov%2Fnchs%2Fnhanes.htm

## Abbreviations

NHANES: National Health and Nutrition Examination Survey
FFQ: food frequency questionnaire
NDI: National Death Index
GED: general educational development
AHEI: Alternative Healthy Eating Index
CPH: Cox proportional-hazards
HRs: hazard ratios
CIs: confidence intervals
Per1: Period 1
SCN: suprachiasmatic nucleus
PTH: parathyroid hormone
CRP: C-reactive protein
