# Supplementary material for: The association of minerals intake in three meals with cancer and all-cause mortality: the U.S. National Health and Nutrition Examination Survey, 2003–2014
Source: BMC Cancer. 2021 Aug 11;21:912. doi: 10.1186/s12885-021-08643-5 (PMC8359108; doi:10.1186/s12885-021-08643-5)
Supplement: Supplementary file 1 — Additional file 1 Supplementary Table 1: The distribution of minerals in each meal. The data in the table is expressed as the mean× 100% (the standard deviation × 100%). [file 12885_2021_8643_MOESM1_ESM.docx]

**Supplementary Table 1** The distribution of minerals in each meal

|  | Breakfast | Lunch | Dinner |
| --- | --- | --- | --- |
| Potassium | 29.42% (19.86%) | 28.91% (23.67%) | 44.83% (27.57%) |
| Calcium | 39.12% (34.66%) | 30.17% (30.60%) | 44.31% (35.90%) |
| Magnesium | 29.25% (19.95%) | 28.29% (23.07%) | 43.18% (26.51%) |
| Copper | 25.96% (20.44%) | 30.22% (37.92%) | 45.35% (42.27%) |
| Sodium | 23.46% (20.39%) | 33.25% (30.70%) | 49.43% (34.07%) |
| Selenium | 25.93% (22.39%) | 31.15% (32.24%) | 50.19% (41.23%) |
| Zinc | 30.80% (31.12%) | 30.13% (30.90%) | 49.47% (41.12%) |
| Phosphorus | 30.19% (21.87%) | 29.96% (25.78%) | 46.50% (29.83%) |
| Iron | 39.22% (38.17%) | 28.10% (26.00%) | 43.76% (32.86%) |

The data in the table is expressed as the mean ×100% (the standard deviation ×100%)
