# Supplementary material for: The association of minerals intake in three meals with cancer and all-cause mortality: the U.S. National Health and Nutrition Examination Survey, 2003–2014
Source: BMC Cancer. 2021 Aug 11;21:912. doi: 10.1186/s12885-021-08643-5 (PMC8359108; doi:10.1186/s12885-021-08643-5)
Supplement: Supplementary file 2 — Additional file 2 Supplementary Table 2: Multivariate adjusted HRs of the total dietary minerals intake with cancer and all-cause mortality [file 12885_2021_8643_MOESM2_ESM.docx]

**Supplementary Table 2** Multivariate adjusted HRs of the total dietary minerals intake with cancer and all-cause mortality

|  | **Cancer Mortality** | | | | **All-cause Mortality** | | | |
| --- | --- | --- | --- | --- | --- | --- | --- | --- |
| **minerals** | **Model 1**^a^ | | **Model 2**^b^ | | **Model 1**^a^ | | **Model 2**^b^ | |
|  | **Case/N** | **HR (95% CI)** | **Case/N** | **HR (95% CI)** | **Case/N** | **HR (95% CI)** | **Case/N** | **HR (95% CI)** |
| **Potassium** |  |  |  |  |  |  |  |  |
| Q1 | 140/5494 | 1 | 140/5494 | 1 | 642/5494 | 1 | 642/5494 | 1 |
| Q2 | 122/5491 | 0.84(0.65-1.09) | 122/5491 | 0.89(0.69-1.17) | 608/5491 | 1.01(0.89-1.14) | 608/5491 | 1.03(0.91-1.17) |
| Q3 | 120/5488 | 0.81(0.61-1.08) | 120/5488 | 0.90(0.67-1.21) | 534/5488 | 0.93(0.81-1.06) | 534/5488 | 0.97(0.84-1.11) |
| Q4 | 117/5492 | 0.76(0.56-1.05) | 117/5492 | 0.88(0.63-1.23) | 492/5492 | 0.88(0.75-1.02) | 492/5492 | 0.93(0.79-1.09) |
| Q5 | 102/5490 | 0.72(0.49-1.04) | 102/5490 | 0.87(0.58-1.30) | 404/5490 | 0.82(0.68-0.98) | 404/5490 | 0.89(0.73-1.08) |
| *P for trend* |  | 0.061 |  | 0.036 |  | 0.512 |  | 0.314 |
| **Calcium** |  |  |  |  |  |  |  |  |
| Q1 | 167/5503 | 1 | 167/5503 | 1 | 720/5503 | 1 | 720/5503 | 1 |
| Q2 | 135/5493 | 0.85(0.68-1.08) | 135/5493 | 0.90(0.71-1.15) | 587/5493 | 0.93(0.83-1.04) | 587/5493 | 0.95(0.84-1.06) |
| Q3 | 108/5482 | 0.73(0.56-0.94) | 108/5482 | 0.79(0.61-1.04) | 542/5482 | 0.96(0.85-1.08) | 542/5482 | 0.99(0.87-1.12) |
| Q4 | 99/5490 | 0.77(0.59-1.02) | 99/5490 | 0.87(0.65-1.17) | 466/5490 | 1.03(0.90-1.17) | 466/5490 | 1.08(0.94-1.24) |
| Q5 | 92/5487 | 0.90(0.67-1.21) | 92/5487 | 1.07(0.77-1.48) | 365/5487 | 1.04(0.90-1.20) | 365/5487 | 1.10(0.94-1.29) |
| *P for trend* |  | 0.222 |  | 0.385 |  | 0.961 |  | 0.116 |
| **Magnesium** |  |  |  |  |  |  |  |  |
| Q1 | 159/5510 | 1 | 159/5510 | 1 | 760/5510 | 1 | 760/5510 | 1 |
| Q2 | 123/5480 | 0.79(0.62-1.02) | 123/5480 | 0.83(0.64-1.07) | 599/5480 | 0.89(0.79-1.00) | 599/5480 | 0.90(0.80-1.02) |
| Q3 | 127/5530 | 0.86(0.65-1.12) | 127/5530 | 0.93(0.70-1.23) | 547/5530 | 0.92(0.80-1.04) | 547/5530 | 0.94(0.82-1.08) |
| Q4 | 107/5463 | 0.74(0.55-1.00) | 107/5463 | 0.83(0.60-1.14) | 443/5463 | 0.80(0.69-0.93) | 443/5463 | 0.84(0.72-0.98) |
| Q5 | 85/5472 | 0.65(0.46-0.93) | 85/5472 | 0.75(0.51-1.11) | 331/5472 | 0.74(0.63-0.88) | 331/5472 | 0.78(0.65-0.94) |
| *P for trend* |  | 0.028 |  | 0.225 |  | 0.001 |  | 0.012 |

^a^Model 1: adjustments included age, sex, ethnicity, income, education level, regular exercise, smoking and drinking status, BMI, prevalence of diabetes, hypertension, hyperlipidemia, nutrient supplement use, AHEI, total daily energy intake.

^b^Model 2: all adjustments in Model 1, and the amount of dietary minerals intake at different time.

Q, Quintile. HR, hazard ratio
