# Supplementary material for: The association of minerals intake in three meals with cancer and all-cause mortality: the U.S. National Health and Nutrition Examination Survey, 2003–2014
Source: BMC Cancer. 2021 Aug 11;21:912. doi: 10.1186/s12885-021-08643-5 (PMC8359108; doi:10.1186/s12885-021-08643-5)
Supplement: Supplementary file 6 — Additional file 6 Supplementary Table 6: Adjusted HRs for the differences in minerals intake between dinner and breakfast and cancer and all-cause mortality by adjusting the minerals intake score instead of individual mineral intake. [file 12885_2021_8643_MOESM6_ESM.docx]

**Supplementary Table 6** Adjusted HRs for the differences in minerals intake between dinner and breakfast and cancer and all-cause mortality by adjusting the minerals intake score instead of individual mineral intake.

|  | Cancer mortality | | All-cause mortality | |
| --- | --- | --- | --- | --- |
|  | Case/N | HR (95%CI) | Case/N | HR (95%CI) |
| **Potassium (breakfast)** | |  |  |  |
| Q1 | 100/5528 | 1 | 415/5528 | 1 |
| Q2 | 112/5516 | 0.97(0.74-1.27) | 538/5516 | 1.17(1.03-1.34) |
| Q3 | 124/5500 | 0.98(0.75-1.29) | 545/5500 | 1.11(0.97-1.26) |
| Q4 | 140/5487 | 1.11(0.85-1.44) | 633/5487 | 1.29(1.13-1.46) |
| Q5 | 125/5424 | 0.99(0.76-1.31) | 549/5424 | 1.17(1.02-1.34) |
| *P* for trend |  | 0.715 |  | 0.011 |
| **Potassium (lunch)** | |  |  |  |
| Q1 | 135/5377 | 1 | 582/5377 | 1 |
| Q2 | 139/5559 | 1.05(0.83-1.34) | 597/5559 | 1.06(0.94-1.19) |
| Q3 | 125/5529 | 0.95(0.74-1.22) | 557/5529 | 1.07(0.95-1.21) |
| Q4 | 91/5508 | 0.70(0.53-0.92) | 454/5508 | 0.90(0.79-1.02) |
| Q5 | 111/5482 | 0.90(0.68-1.18) | 490/5482 | 1.10(0.97-1.26) |
| *P* for trend |  | 0.054 |  | 0.740 |
| **Potassium (dinner)** | |  |  |  |
| Q1 | 139/5293 | 1 | 601/5293 | 1 |
| Q2 | 117/5533 | 0.82(0.64-1.06) | 563/5533 | 0.97(0.86-1.09) |
| Q3 | 123/5540 | 0.83(0.65-1.08) | 514/5540 | 0.91(0.80-1.03) |
| Q4 | 114/5552 | 0.72(0.55-0.94) | 492/5552 | 0.92(0.81-1.05) |
| Q5 | 108/5537 | 0.74(0.56-0.98) | 507/5537 | 0.87(0.84-1.11) |
| *P* for trend |  | 0.020 |  | 0.409 |
| **Calcium (breakfast)** | |  |  |  |
| Q1 | 104/5556 | 1 | 465/5556 | 1 |
| Q2 | 128/5539 | 1.15(0.88-1.49) | 493/5539 | 1.02(0.90-1.16) |
| Q3 | 116/5506 | 1.03(1.78-1.34) | 571/5506 | 1.14(1.01-1.29) |
| Q4 | 131/5457 | 1.11(0.85-1.44) | 613/5457 | 1.19(1.05-1.35) |
| Q5 | 122/5397 | 1.15(0.88-1.51) | 538/5397 | 1.21(1.06-1.37) |
| *P* for trend |  | 0.448 |  | 0.000 |
| **Calcium (lunch)** | |  |  |  |
| Q1 | 135/5370 | 1 | 591/5370 | 1 |
| Q2 | 133/5574 | 1.00(0.78-1.27) | 584/5574 | 1.03(0.92-1.16) |
| Q3 | 132/5507 | 0.98(0.76-1.25) | 527/5507 | 0.98(0.86-1.10) |
| Q4 | 96/5503 | 0.79(0.61-1.04) | 516/5503 | 1.06(0.93-1.20) |
| Q5 | 105/5501 | 0.94(0.72-1.23) | 462/5501 | 1.12(0.98-1.28) |
| *P* for trend |  | 0.258 |  | 0.104 |
| **Calcium (dinner)** | |  |  |  |
| Q1 | 142/5327 | 1 | 619/5327 | 1 |
| Q2 | 123/5553 | 0.85(0.67-1.09) | 554/5553 | 0.93(0.82-1.05) |
| Q3 | 130/5537 | 0.89(0.69-1.14) | 564/5537 | 1.00(0.89-1.13) |
| Q4 | 114/5529 | 0.79(0.61-1.03) | 506/5529 | 0.95(0.84-1.07) |
| Q5 | 92/5509 | 0.71(0.53-0.94) | 437/5509 | 0.94(0.82-1.07) |
| *P* for trend |  | 0.018 |  | 0.519 |
| **Magnesium (breakfast)** | |  |  |  |
| Q1 | 99/5585 | 1 | 437/5585 | 1 |
| Q2 | 115/5564 | 1.06(0.81-1.40) | 499/5564 | 1.02(0.89-1.17) |
| Q3 | 133/5409 | 1.20(0.92-1.75) | 601/5409 | 1.27(1.12-1.45) |
| Q4 | 137/5473 | 1.14(0.87-1.49) | 602/5473 | 1.21(1.07-1.38) |
| Q5 | 117/5424 | 1.05(0.79-1.39) | 541/5424 | 1.17(1.03-1.34) |
| *P* for trend |  | 0.649 |  | 0.002 |
| **Magnesium (lunch)** | |  |  |  |
| Q1 | 136/5403 | 1 | 600/5403 | 1 |
| Q2 | 135/5555 | 1.02(0.80-1.30) | 618/5555 | 1.07(0.95-1.20) |
| Q3 | 126/5514 | 0.94(0.73-1.20) | 529/5514 | 0.96(0.85-1.09) |
| Q4 | 103/5532 | 0.79(0.60-1.03) | 504/5532 | 0.99(0.88-1.13) |
| Q5 | 101/5451 | 0.87(0.66-1.14) | 429/5451 | 0.99(0.86-1.13) |
| *P* for trend |  | 0.100 |  | 0.638 |
| **Magnesium (dinner)** | |  |  |  |
| Q1 | 145/5367 | 1 | 648/5367 | 1 |
| Q2 | 123/5501 | 0.85(0.67-1.09) | 568/5501 | 0.95(0.84-1.07) |
| Q3 | 115/5508 | 0.76(0.59-0.99) | 528/5508 | 0.89(0.79-1.00) |
| Q4 | 113/5544 | 0.76(0.58-0.99) | 504/5544 | 0.91(0.80-1.03) |
| Q5 | 105/5535 | 0.72(0.54-0.96) | 432/5535 | 0.86(0.75-0.98) |
| *P* for trend |  | 0.016 |  | 0.027 |
| **Copper (breakfast)** | |  |  |  |
| Q1 | 78/4935 | 1 | 374/4935 | 1 |
| Q2 | 140/6185 | 1.30(0.98-1.72) | 587/6185 | 1.10(0.97-1.26) |
| Q3 | 152/6219 | 1.26(0.95-1.67) | 676/6219 | 1.19(1.04-1.36) |
| Q4 | 143/6384 | 1.18(0.88-1.57) | 692/6384 | 1.22(1.07-1.39) |
| Q5 | 88/3732 | 1.29(0.94-1.78) | 351/3732 | 1.17(1.07-1.37) |
| *P* for trend |  | 0.371 |  | 0.008 |
| **Copper (lunch)** | |  |  |  |
| Q1 | 77/2899 | 1 | 325/2899 | 1 |
| Q2 | 116/4700 | 1.02(0.76-1.36) | 501/4700 | 1.04(0.90-1.20) |
| Q3 | 166/7316 | 0.97(0.74-1.28) | 717/7316 | 1.03(0.90-1.18) |
| Q4 | 141/7634 | 0.78(0.58-1.04) | 712/7634 | 1.04(0.90-1.19) |
| Q5 | 101/4906 | 0.87(0.63-1.19) | 425/4906 | 1.03(0.88-1.20) |
| *P* for trend |  | 0.071 |  | 0.744 |
| **Copper (dinner)** | |  |  |  |
| Q1 | 91/3601 | 1 | 409/3601 | 1 |
| Q2 | 142/6002 | 0.97(0.74-1.27) | 637/6002 | 1.03(0.90-1.17) |
| Q3 | 149/7670 | 0.77(0.59-1.02) | 611/7670 | 0.90(0.79-1.02) |
| Q4 | 135/6260 | 0.83(0.62-1.01) | 697/6260 | 1.03(0.90-1.18) |
| Q5 | 84/3922 | 0.80(0.58-1.11) | 326/3922 | 0.90(0.76-1.05) |
| *P* for trend |  | 0.089 |  | 0.289 |

Adjustments included age, sex, ethnicity, income, education level, regular exercise, smoking and drinking status, BMI, prevalence of diabetes, hypertension, hyperlipidemia, nutrient supplement use, AHEI, total daily energy intake and minerals intake score. Q, Quintile. HR, hazard ratio.
