# Supplementary material for: The association of minerals intake in three meals with cancer and all-cause mortality: the U.S. National Health and Nutrition Examination Survey, 2003–2014
Source: BMC Cancer. 2021 Aug 11;21:912. doi: 10.1186/s12885-021-08643-5 (PMC8359108; doi:10.1186/s12885-021-08643-5)
Supplement: Supplementary file 7 — Additional file 7 Supplementary Table 7: The food sources of the minerals. The data in the table are expressed as median (minimum, maximum). [file 12885_2021_8643_MOESM7_ESM.docx]

**Supplementary Table 7** The food sources of the minerals.

| Classification | Food  Name | Note | Potassium | Calcium | Magnesium | Copper |
| --- | --- | --- | --- | --- | --- | --- |
| Dairy | Cheese | (cup eq.) | 114.00  (0.00,4439.00) | 232.00  (0.00,4304.50) | 17.00 (0.00,429.00) | 0.07  (0.00,3.19) |
|  | Milk | Fluid milk and calcium fortified soy milk (cup eq.) | 435.00  (0.00,5950.50) | 260.50  (0.00,5013.00) | 41.00 (0.00,652.00) | 0.13  (0.00,14.42) |
|  | Yogurt | (cup eq.) | 0.00  (0.00,3209.00) | 0.00  (0.00,1568.00) | 0.00 (0.00,251.00) | 0.00  (0.00,1.02) |
|  | Total | Total milk, yogurt, cheese, and whey  (cup eq.) | 612.00  (0.00,6393.50) | 457.50  (0.00,5737.00) | 63.00 (0.00,854.00) | 0.22  (0.00,14.42) |
| Fruit | Citmlb | Intact fruits (whole or cut) of citrus, melons, and berries  (cup eq.) | 0.00  (0.00,3242.00) | 5.00  (0.00,1292.00) | 0.00 (0.00,283.00) | 0.19  (0.00,1.19) |
|  | Juice | Fruit juices, citrus and non citrus  (cup eq.) | 12.00  (0.00,12000.00) | 12.00  (0.00,7976.00) | 1.00 (0.00,800.00) | 0.03  (0.00,2.72) |
|  | Other | Intact fruits (whole or cut); excluding citrus, melons, and berries (cup eq.) | 174.50  (0.00,4290.00) | 12.00  (0.00,4494.00) | 12.00 (0.00,710.00) | 0.09  (0.00,4.84) |
|  | Total | Total intact or cut fruits and fruit juices (cup eq.) | 402.00  (0.00,7063.00) | 42.00  (0.00,7976.00) | 29.50 (0.00,710.00) | 0.14  (0.00,4.84) |
| Grain | Whole | Whole grains  (oz. eq.) | 96.00  (0.00,2614.00) | 46.00  (0.00,3975.00) | 26.00 (0.00,762.00) | 0.11  (0.00,2.60) |
|  |  |  |  |  |  |  |
|  | Refined | Refined or non-whole grains  (oz. eq.) | 544.50  (0.00,4489.50) | 233.00  (0.00,3177.00) | 80.00 (0.00,671.00) | 0.39  (0.00,13.71) |
|  | Total | Total whole and refined grains  (oz. eq.) | 598.00  (0.00,4375.50) | 259.00  (0.00,4086.00) | 95.50 (0.00,699.00) | 0.44  (0.00,13.71) |
| PF | Cured meat | Cured/luncheon meat made from beef, pork, or poultry  (oz. eq.) | 112.00  (0.00,7235.00) | 6.50  (0.00,5441.00) | 6.50 (0.00,379.00) | 0.49  (0.00,3.19) |
|  | Eggs | Eggs (chicken, duck, goose, quail) and egg substitutes  (oz. eq.) | 178.00  (0.00,4034.00) | 63.00  (0.00,2909.00) | 19.00 (0.00,501.00) | 0.10  (0.00,26.55) |
|  | Meat | Beef, veal, pork, lamb, game meat; excludes organ meats and cured | 297.00  (0.00,6605.00) | 33.00  (0.00,3295.00) | 23.00 (0.00,589.00) | 0.12  (0.00,4.27) |
|  | Nutsds | Peanuts, tree nuts, and seeds, excludes coconut  (oz. eq.) | 0.00  (0.00,4701.00) | 7.00  (0.00,2223.00) | 0.00 (0.00,1290.00) | 0.46  (0.00,8.59) |
|  | Organ | Organ meat from beef, veal, pork, lamb, game, and poultry (oz. eq.) | 0.00  (0.00,2592.00) | 0.00  (0.00,420.00) | 0.00 (0.00,210.00) | 0.00  (0.00,49.19) |
|  | Poult | Chicken, turkey, Cornish hens, and game birds; excludes organ meats | 161.00  (0.00,7576.00) | 14.50  (0.00,2951.00) | 16.00 (0.00,475.00) | 0.06  (0.00,4.88) |
|  | Seafood low | Seafood (finfish, shellfish and other seafood) low in n-3 fatty acids  (oz. eq.) | 0.00  (0.00,4256,00) | 0.00  (0.00,2430.00) | 0.00 (0.00,637.00) | 0.00  (0.00,17.81) |
|  | Seafood high | Seafood (finfish, shellfish and other seafood) high in n-3 fatty acids  (oz. eq.) | 0.00  (0.00,4768.00) | 0.00  (0.00,2166.00) | 0.00 (0.00,398.00) | 0.00  (0.00,6.21) |
|  | Soy | Soy products, excluding calcium fortified soy milk and immature | 0.00  (0.00,4993.00) | 0.00  (0.00,1905.00) | 0.00 (0.00,1882.00) | 0.00  (0.00,11.45) |
|  | MPS total | Total meat, poultry, seafood, organ meats, and cured meat (oz. eq.) | 623.00  (0.00,8668.00) | 87.00  (0.00,2912.00) | 53.00 (0.00,468.00) | 0.24  (0.00,36.56) |
|  | Total | Total meat, poultry, seafood, organ meats, cured meat, eggs, soy, and nuts and seeds; excludes legumes  (oz. eq.) | 790.50  (0.00,8774.00) | 164.00  (0.00,2948.00) | 80.00 (0.00,1229.00) | 0.38  (0.00,36.61) |
| Solid | Fats | Solid fats (grams) | 1048.00  (0.00,8364.00) | 473.50  (0.00,5737.00) | 107.50 (0.00,968.00) | 0.46  (0.00,36.11) |
| Vegetable | Drkgr | Dark green vegetables  (cup eq.) | 0.00  (0.00,4610.00) | 0.00  (0.00,1358.00) | 0.00 (0.00,656.00) | 0.00  (0.00,2.05) |
|  | Legumes | Legumes computed as vegetables (cup eq.) | 0.00  (0.00,6386.00) | 0.00  (0.00,1871.00) | 0.00 (0.00,589.00) | 0.00  (0.00,2.71) |
|  | Redor tomato | Tomatoes and tomato products  (cup eq.) | 359.00  (0.00,6661.00) | 59.50  (0.00,3433.00) | 28.00 (0.00,1002.00) | 0.17  (0.00,5.08) |
|  | Redor other | Other red and orange vegetables, excluding tomatoes and tomato products  (cup eq.) | 0.00  (0.00,7576.00) | 11.00  (0.00,2166.00) | 0.00 (0.00,973.00) | 0.01  (0.00,4.92) |
|  | Redor total | Total red and orange vegetables (tomatoes + other red and orange)  (cup eq.) | 434.50  (0.00,5776.00) | 71.50  (0.00,3433.00) | 35.00 (0.00,1003.00) | 0.19  (0.00,5.08) |
|  | Starchy potato | White potatoes (cup eq.) | 256.00  (0.00,7576.00) | 11.00  (0.00,5429.00) | 15.00 (0.00,972.00) | 0.11  (0.00,4.92) |
|  | Starchy other | Other starchy vegetables, excluding white potatoes (cup eq.) | 0.00  (0.00,7576.00) | 0.00  (0.00,1257.00) | 0.00 (0.00,1882.00) | 0.00  (0.00,9.62) |
|  | Starchy total | Total starchy vegetables (white potatoes + other starchy)  (cup eq.) | 333.00  (0.00,8184.00) | 17.00  (0.00,5429.00) | 22.00 (0.00,972.00) | 0.13  (0.00,4.92) |
|  | Other | Other vegetables not in the vegetable components listed above (cup eq.) | 434.50  (0.00,21201.50) | 79.50  (0.00,3634.50) | 38.00 (0.00,1520.50) | 0.20  (0.00,27.12) |
|  | Total | Total dark green, red and orange, starchy, and other vegetables; excludes legumes  (cup eq.) | 812.00  (0.00,21879.50) | 128.00  (0.00,3762.50) | 64.50 (0.00,1585.00) | 0.33  (0.00,27.12) |

The data in the table are expressed as median (minimum, maximum)
